# Epidural Volume of Injectate Using a Dose Regimen Based on Occipito-Coccygeal Spinal Length (OCL): Randomized Clinical Study Comparing Different Ropivacaine Concentrations, with or without Morphine, in Bitches Undergoing Total Unilateral Mastectomy

**DOI:** 10.3390/ani12050587

**Published:** 2022-02-25

**Authors:** Hamaseh Tayari, Pablo E. Otero, Marco D’Agostino, Flavia Bartolini, Angela Briganti

**Affiliations:** 1 Southern Counties Veterinary Specialists (SCVS), Forest Corner Farm, Hangersley, Ringwood, Hampshire BH24 3JW, UK; 2 Department of Anesthesiology and Pain Management, Facultad de Ciencias Veterinarias, Universidad de Buenos Aires, Buenos Aires C1427CWO, Argentina; potero@fvet.uba.ar; 3 Department of Veterinary Sciences, University of Pisa, 56124 Pisa, Italy; marco21d@hotmail.it (M.D.); flaviabartolini@gmail.com (F.B.); angela.briganti@unipi.it (A.B.)

**Keywords:** epidural, locoregional, anaesthesia, dog, ropivacaine, occipito-coccygeal length, morphine, mastectomy

## Abstract

**Simple Summary:**

Radical mastectomy involves the removal of mammary tissue and the surrounding lymph nodes. Inadequate perioperative analgesia for such extensive tissue resection may result in adaptive and maladaptive pain. The influence of anaesthetic/analgesic protocols on tumour progression and recurrence has been previously demonstrated, particularly the value of opioid-reduction strategies using locoregional and neuraxial anaesthesia has emerged. The aims of this study were (I) to evaluate the efficacy of lumbosacral epidural anaesthesia using a total volume of local anaesthetic tailored on individual spinal length; (II) to compare different concentrations of ropivacaine, with or without morphine, in terms of intraoperative inhalant anaesthetic and perioperative systemic opioids requirements; (III) to evaluate the incidence of intraoperative cardiorespiratory side effects, postoperative motor, and urinary functions impairments. The results of this study showed a significant reduction of perioperative opioid requirement and lower cardiorespiratory complications in dogs receiving epidural anaesthesia, with a volume injected based on the individual spinal length. Further studies comparing the volume of epidural injectate calculated based on bodyweight or on the individual spinal length are needed.

**Abstract:**

A prospective, randomized clinical trial was designed to compare four epidural treatments in dogs undergoing total unilateral mastectomy. The epidural volume of injectate was based on the individual occipito-coccygeal length (OCL) aiming to reach the first thoracic vertebra (T_1_). The first ten dogs were allocated in a control group (C) and did not receive epidural treatment. Subsequently, forty dogs were randomly allocated in four groups of ten: epidural ropivacaine 0.5% (R_0.5%_); morphine 0.1 mg kg^−1^ plus ropivacaine 0.5% (MR_0.5%_); morphine 0.1 mg kg^−1^ plus ropivacaine 0.35% (MR_0.35%_); morphine 0.1 mg kg^−1^ plus ropivacaine 0.25% (MR_0.25%_). Intraoperatively, isoflurane requirement (1.3% vs. <1.1% FE’Iso) and fentanyl requirement (9.8 vs. <1.1 µg kg^−1^ h^−1^) were significantly higher in C group compared to all epidural groups. Postoperatively, methadone requirement was higher (1.8 mg kg^−1^ vs. <0.8 mg kg^−1^) for C group compared to all epidural treatment groups. The ability to walk and to urinate returned 4 h earlier in MR_0.35%_ and MR_0.25%_. The mean epidural volume of ropivacaine, using a dose regimen based on OCL, to reach T_1_ was about 0.15 mL cm^−1^. The addition of morphine further reduced the methadone requirement, without affecting urinary and motor functions.

## 1. Introduction

Mammary gland neoplasia is one of the most frequently diagnosed neoplasia in dogs, accounting for up to 42% of all tumours in female dogs and with an incidence of 50% of malignancies. Despite efforts to develop treatments, total unilateral mastectomy remains the first choice [1,2]. This surgical treatment requires extensive tissue resection for which inadequate perioperative analgesia may result in acute and persistent postoperative pain [3,4].

In the last several decades, the scientific community investigated the influence of anaesthetic and analgesic techniques on tumours progression and recurrence; particularly, the value of opioid sparing strategies obtained using locoregional techniques, and neuraxial anaesthesia has emerged [5,6,7,8,9]. Epidural analgesia, may provide pre-emptive analgesia by inhibiting central sensitization and modulating afferent signals to the dorsal horn, reducing intraoperative inhalant agent and perioperative systemic opioid requirements [10,11]. Furthermore, a single-dose neuraxial opioid added to the local anaesthetic (LA) has been shown to enhance postoperative analgesic efficacy and prolong duration of action of the LA [10,11,12,13].

On the other hand, hypotension, motor paralysis, and urinary retention are possible complications reported after neuraxial anaesthesia.; however, if differential blockade strategies are used (administration of a less lipophilic and less concentrated LA), the incidence of these adverse effects can be reduced [14,15,16]. The “differential” blockade effect is attributed to a dissimilar susceptibility in larger and more myelinated Aα motor nerve fibers when compared with Aδ and C fibers to LA [17].

When performing lumbosacral epidural anaesthesia, two methods are commonly used to determine the volume of injectate: one based on body weight, and another based on the vertebral column length [18,19]. Interestingly, controversies regarding the most appropriate method are still ongoing [20,21]. In case the volume is calculated using a dose regimen based on the vertebral column length, the volume is obtained either by using a fixed volume per cm of vertebral column length, as 1 mL per 10 cm^−1^ of occipito-coccygeal length (OCL) [12,21] or by using a nomogram that allows adjusting the volume of injectate as a function of the desired percentage of OCL to desensitize [22]. In detail, the OCL is measured from the occipital bone to the first coccygeal vertebra; then, the desired length of the column to anaesthetize is measured and calculated as a percentage of the total OCL (OCL%). Applying these variables to the nomogram, the total volume to be injected is easily obtained [22].

The aims of the present study were (I) to evaluate the efficacy of a lumbosacral epidural anaesthesia obtained with a volume of injectate (ropivacaine) calculated using a dose regimen of LA based on OCL% targeted to reach the first thoracic vertebra (T_1_), (II) to compare the analgesic efficacy obtained using different ropivacaine concentrations, with or without morphine, and (III) to determine the incidence of cardiovascular and respiratory adverse effects after epidural injection of LA volume based on OCL and OCL% in dogs.

For this purpose, in bitches undergoing total unilateral mastectomy, four epidural treatments, using different ropivacaine concentrations (± morphine) used as the analgesic plan were compared. In all protocols, the volume epidurally injected was calculated based on the individual OCL aiming to reach T_1_. Intraoperative and postoperative opioids requirements and motor and urinary function were assessed hourly for twenty-four hours in all dogs studied.

## 2. Materials and Methods

### 2.1. Animals

The study was conducted in compliance with the European Welfare Act and with the approval of the local Ethical Committee of University of Pisa (*n* 32/2014). The study was conducted at the Veterinary Teaching Hospital of Pisa and a written informed consent was obtained from the owners before enrolling the dogs in this study. Based on clinical examination, only female dogs assigned to American Society of Anesthesiologists (ASA) category I to III, undergoing total unilateral mastectomy were included. Exclusion criteria were: ASA status > III, body condition score (BCS) < 3/9 or BCS > 7/9 [23], preoperative opioid administration, nervous or aggressive temperament, pre-existing pain, skin infections, neurological or neuromuscular disease, coagulopathy (defined by <80,000 platelets μL^−1^, or a Prothrombin Time(PT) or Partial Thromboplastin Time (PTT) > 50% above the normal laboratory reference interval, or multiple surgical procedures.

The study was divided into two phases. First, ten dogs undergoing total unilateral mastectomy, who did not receive neuraxial anaesthesia, were enrolled as controls (group C). In this group, systemic perioperative opioids and intraoperative inhalant anaesthetic requirement, time to resumption of voluntary locomotor activity, and voluntary urination were monitored. In the second phase, 40 dogs undergoing total unilateral mastectomy were randomly assigned using an online software (https://www.random.org, accessed on 1 September 2019) to one of the following groups: epidural ropivacaine (Naropina, 5 mg mL^−1^,AstraZeneca, Italy) 0.5% group (R_0.5%_); morphine 0.1 mg kg^−1^ (morphine preservative-free 10 mg mL^−1^) plus ropivacaine 0.5% group (MR_0.5%_); morphine 0.1 mg kg^−1^ plus ropivacaine 0.35% group (MR_0.35%_); morphine 0.1 mg kg^−1^ plus ropivacaine 0.25% group (MR_0.25%_). Systemic perioperative opioid requirement, intraoperative inhalant anaesthetic requirement, timing to resumption of voluntary locomotor activity, and voluntary urination were evaluated.

A sample size calculation was performed using values pertinent to the intraoperative fentanyl requirement with an α error of 0.05, and a β error of 0.2, considering a mean fentanyl infusion of 5 mcg kg^−1^ h as the maximum endpoint, in order to identify the minimum number of dogs required per group, which resulted in six. The null hypothesis was considered if dogs received more than 2 mcg kg^−1^ h^−1^ [24].

### 2.2. Anaesthetic Management

Food, but not water, was withheld for 8 h prior to surgery. All dogs were premedicated intramuscularly (IM) with morphine (0.3 mg kg^−1^). After 20 min, an intravenous catheter was placed in one of the cephalic veins and lactated Ringer’s solution at 5 mL kg^−1^ h^−1^ started. Approximately 20 min later, induction of anaesthesia was achieved with propofol (Propovet multidose 10 mg mL^−1^, Zoetis, Roma, Italy) intravenously (IV) titrated to effect. Orotracheal intubation was performed to maintain anaesthesia with isoflurane (IsoFlo FL 250; ESTEVE SpA, Milan, Italy) vaporized in 100% oxygen, fractional inspired oxygen (FiO_2_), and delivered through a rebreathing system. A 22- or 20-gauge catheter (Biovalve Safe, Vygon, Padova, Italy) was placed in one of the dorsal pedal arteries to measure invasive blood pressure (IBP). Routine anaesthetic monitoring, heart rate (HR), respiratory rate, IBP, end-tidal carbon dioxide (PE’CO_2_), end-tidal isoflurane (FE’Iso), and peripheral arterial oxygen saturation (SpO_2_) were continuously monitored and recorded every five minutes and at defined surgical time points (Table 1) using a multiparameter monitor (S5 Compact Anaesthesia Monitor; Datex Ohmeda, Louisville, KY, USA). For IBP, a transducer positioned and zeroed at the level of the sternum was used. Parameters registered five minutes before the start of the surgery were recorded as S_base_ and considered as baseline. At S_base_ time, FE’Iso was set to 1.3% and subsequently decreased every 5–10 min, to obtain decremental steps of 0.05% of the FE’Iso values. Isoflurane was reduced only if the recorded physiological parameters remained within 20% of baseline [25] while maintaining a surgical anaesthetic plane, defined as a patient with absent palpebral reflex, mild jaw tone, and lack of purposeful movement [26].

A rise in HR or mean arterial blood pressure (MAP) values of 20% or more compared to S_base_ values, was evaluated as nociception and rescue analgesia as intraoperative fentanyl (Fentadon, 50 mcg mL^−1^, Eurovet Animal Health B.V., Bladel, The Netherlands) was administered as follows: up to two boluses of 1 µg kg^−1^ IV and in the case of unrestored parameters as infusion starting at 2.5 µg kg^−1^ h^−1^ and then titrated to return to the S_base_ values recorded before the beginning of the surgery. The total amount of intraoperative fentanyl (boluses and or infusion) was calculated by dividing the total amount of fentanyl administered by the duration of the surgery (h) and then by the bodyweight of the patient. Mechanical ventilation was commenced in the event of a PE’CO_2_ ≥ 50 mmHg (6.6 kPa) with a volume control mode ventilation (Datex-Ohmeda 7900 SmartVent, GE Healthcare, Bensalem, PA, USA), with inspiratory:expiratory ratio between 1:2.5 and 1:3, with an appropriate tidal volume and respiratory rate adjusted based on patient requirements aimed to maintain an PE’CO_2_ between 35 to 50 mmHg (4.6 to 6.6 kPa). In case of hypotension (MAP less than 60 mmHg), this was pharmacologically treated with atropine 20 mcg kg^−1^ IV (Atropina Solfato; ATI, Naples, Italy) or dopamine (5–10 µg kg^−1^ min^−1^) (Hospira; Hospira, Naples, Italy) where appropriate. During the surgery, all animals were placed on an actively warming air circulating-blanket system (Bair Hugger 505 warmer unit) to reduce the risk of hypothermia.

At the end of the surgery and before switching off the isoflurane vaporizer, for each patient, a rigid urinary catheter was used to empty the bladder; animals were then moved to the recovery area and extubated once able to swallow. The same two investigators (FB and MDA), blinded to the group assignment, followed all postoperative monitoring and recorded all the parameters. The same two surgeons performed all surgical interventions. Total surgery time, considered as the time from skin incision (SX_1_) to skin closure (SX_5_), and the time between epidural execution and the end of the surgery, were recorded.

### 2.3. Epidural Administration

**Figure 1:**
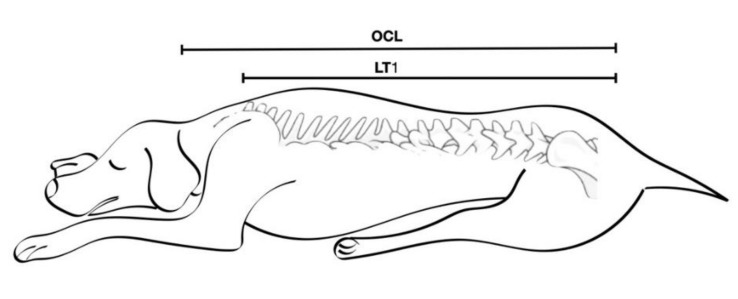
Representative measurements of occipital-coccygeal length (OCL) and the length from the coccygeal vertebra to the first thoracic vertebra (LT_1_).

After induction of anaesthesia, all dogs were positioned in sternal recumbency with the pelvic limbs pulled forward. The OCL was measured from occipital bone to the first coccygeal vertebra. The desired extension of the epidural block was determined by measuring the length from the first coccygeal to the first thoracic vertebra (LT_1_) (Figure 1). All measurements were made using a standard meter.

The OCL% length was estimated as a percentage of the OCL using the following equation:

OCL% = [100 × LT_1_] (cm)/[OCL] (cm) (1)

Applying OCL and OCL% in a previously published nomogram [22,27], the total volume of ropivacaine to inject was obtained. Morphine (Morfina Cloridrato, 10 mg mL^−1^, Molteni, Italy) 0.1 mg kg^−1^ was added to the final volume of injectate in all dogs belonging to the following groups MR_0.5%_, MR_0.35%_, and MR_0.25%_.

In all dogs, including those assigned to C group, the L_7_–S_1_ area was shaved and clipped to perform the epidural. In all dogs except for those in the C group, the skin of the lumbar–sacral region was aseptically prepared and the epidural performed at L_7_–S_1_ level using an epidural Tuohy’s needle and a loss of resistance syringe (Vygon, Padova, Italy) filled with approximately 1 mL of air. The Tuohy needle was advanced until the interspinous ligament and the ligamentum flavum were pierced. At this point, the Tuohy stylet was removed and the loss of resistance syringe was connected. While the Tuohy’s needle was advanced with one hand, with the index finger of the other hand, the plunger was constantly pushed to check the resistance toward the epidural space. Correct placement of the needle was confirmed by the presence of a distinct “popping sensation”, as a result of needle penetration into the ligamentum flavum and by the loss of resistance to injection of a maximum of 0.5 mL of air. Before injection, the absence of cerebrospinal fluid or blood in the needle hub was checked. Once the correct position of the tip of the needle was confirmed, the low resistance syringe was removed and replaced by the syringe containing the drugs, aseptically prepared. The epidural injection was delivered at a rate of approximately 2 mL/min.

The abolition of the patellar ligament reflex (Westphal’s sign) was assessed 5 min later using a standard Taylor’s reflex hammer. The same investigator (H.T.) blinded to the syringes content, prepared by a different investigator (A.B.), performed all the epidural injections; the correct execution of all epidural techniques was assessed by an expert supervisor (A.B.).

### 2.4. Postoperative Assessment

After extubation, 2 mg kg^−1^ of carprofen (Rimadyl, Zoetis, Roma, Italy) was administered subcutaneously (SC )in all dogs.

Pain was assessed postoperatively each hour for 24 h, starting from 1 h after spontaneous head lifting (T1), using the Short-Form Glasgow Composite Measure Pain Scale (SF-GCMPS) [28] by two investigators Flavia Bartolini (F.B.) and Marco D’Agostino (M.D.), trained in the use of the scale and unaware of group assignment.

Postoperative rescue analgesia with methadone (Semfortan, 10 mg mL^−1^, Dechra, Italy) 0.2 mg kg^−1^ IV was provided if SF-GCMPS resulted ≥ 5. Elapsed time from the epidural execution to first rescue analgesia treatment was recorded as analgesic duration.

Besides the postoperative pain evaluation, cutaneous trunci muscle (CTM) reflex, bilateral patellar reflex, and bilateral withdrawal response were also evaluated starting from the extubation time every 15 min for the first hour and then hourly, until each reflex was restored. The CTM reflex was elicited by pinching the skin 2 cm from the midline bilaterally at the level of the caudal border of the scapulae, thirteen thoracic (T_13_), third lumbar (L_3_), and L_6_ levels looking for contraction of the CTM bilaterally [29]. Patellar reflex was elicited by tapping the patella ligament and looking for a quick extension of the stifle, by pinching the skin of the foot and looking for flexion of the hock.

Among the reflexes, the ability to stand and/or to walk was also recorded when present. Last, the first spontaneous urination episode or urinary retention (defined as failure to urinate within the first 24 h) were recorded.

A month follow-up period was planned to evaluate any neurological deficits or side effects, such as skin reaction, pruritus, and pain at the injection site.

### 2.5. Statistical Analysis

Data distribution was analysed with a D’Agostino and Person test. Parametric data were expressed as mean and standard deviation (SD), while nonparametric data were expressed as median and range. One-way analysis of variance for repeated data (ANOVA) with a Tukey test as post hoc was used to evaluate the parameters recorded over time for each group. Friedman’s test was used for the statistical evaluation of the postoperative pain scale score. One-way analysis of variance (ANOVA) was used to evaluate differences among groups for the clinical parameters examined at each time interval, to compare the dose of propofol required for the induction and isoflurane required for the maintenance of general anaesthesia to compare total fentanyl and methadone dose required. Values for *p* < 0.05 were considered significant.

## 3. Results

Breed distribution, age, and weight are reported in Table 2 and Table 3.

There were no significant differences between groups in respect to age, weight, baseline physiological parameters, dose of propofol required for induction of anaesthesia, duration of anaesthesia, and surgery.

All fifty dogs completed the intraoperative phase of the clinical study; however, two dogs belonging to group C failed the protocol during the postoperative period. Both of these dogs required additional treatment with ketamine infusion to control postoperative pain and one required dexmedetomidine infusion to control signs of anxiety. Anxiety, fear, and attempt to bite were observed in 70% of the animals belonging to C group compared to none recorded in the other groups. In one dog belonging to the MR_0.35%_ group, the protocol failed during the postoperative period as it required a revision surgery, due to a postoperative haemorrhage; it was therefore excluded from all the postoperative evaluations.

General anaesthesia was obtained with a mean dose of propofol of 4.5 ± 2 mg kg^−1^. Time elapsed between the epidural execution and the beginning of the surgery resulted as 46 ± 10 min, surgery time (SX_1_–SX_5_) was 98 ± 21 min, and the time between the epidural execution and the end of surgery was 120 ± 15 min, with no significant differences between the groups.

There were no significant differences between groups for HR, respiratory rate (fR), MAP, PE′CO_2_ values, and temperature (Table 4); however, 10/10 dogs in C group compared to the 4/40 dogs belonging to epidural protocols (two in R_0.5%_ and two in MR_0.5%_) required mechanical ventilation to maintain normocapnia. Significant differences, *p*-Value < 0.00001 (significant at *p* < 0.05) between the C and R_0.5%_ groups in regard of FE’Iso was recorded (1.3 ± 0.1% for C group and 1.1 ± 0.04% for R_0.5%_), but no significant differences between epidural groups for the mean value of FE’Iso was recorded (Table 4).

During the surgery, 5 of the 10 dogs in C group required cardiovascular support; 4 dogs received dopamine at 5–8 µg kg^−1^ min^–1^ (mean time of administration 30 ± 17 min; mean MAP 65 ± 8 mmHg) to manage hypotension, and 1 required atropine IV to manage concomitant bradycardia (<50 bpm) presented at the end of the surgery (dopamine was contextually discontinued). One dog in R_0.5%_ and one in MR_0.5%_ received dopamine at 5 µg kg^−1^ min^−1^ for 20 and 35 min at the beginning of the surgery (S_X1_ to S_X2_) to restore the MAP. For all groups, the mean length of OCL, LT_1_, OCL%, and the volume injected are reported in Table 3.

All dogs in group C required fentanyl infusion for the entire surgical procedure starting from S_X1_. Intraoperative fentanyl requirement was significantly higher in group C (9.8 ± 5.5 µg kg^−1^ h^−1^), compared to all other groups. No dogs in R_0.5%_ required fentanyl infusion; however, in 1/10 dogs, two boluses of 1 µg kg^−1^ were required, for a total of 1.1 ± 0.5 µg kg^−1^ h^−1^. Any of the dogs that received epidural morphine required intraoperative fentanyl infusion; however, fentanyl boluses were needed to restore cardiovascular parameters for a total amount as 0.2 ± 0.14 µg kg^−1^ h^−1^ for MR_0.5%_ (3/10), 0.7 ± 0.6 µg kg^−1^ h^−1^ for MR_0.35%_ (2/10), and 1.2 ± 0.9 µg kg^−1^ h^−1^ for MR_0.25%_ (2/10).

All dogs in group C required postoperative rescue analgesia one hour after endotracheal extubation, and the total methadone requirement in the first 24 h was significantly higher compared to all the other groups. In MR_0.5%_, methadone requirement was significantly lower in comparison to all other groups (Table 5) and no dog needed methadone within the first 24 h. The average timing for postoperative rescue analgesia was at 18 and at 12 h from epidural for MR_0.35%_ and MR_0.25%_ groups, respectively. The total postoperative methadone administered over 24 h was 1.8 ± 0.5 mg kg^−1^ in C group, 0.8 ± 0.1 mg kg^−1^ in R_0.5%_, 0 mg kg^−1^ in MR_0.5%_, 0.3 ± 0.05 mg kg^−1^ in MR_0.35%_, and 0.4 ± 0.03 mg kg^−1^ in MR_0.25%_ (Table 5).

Resumption of locomotor activity was significantly quicker in group C compared to all other groups. Within the groups of dogs who received epidural LA, significantly quicker recovery of locomotor activity was recorded in MR_0.25%_ and MR_0.35%_ groups compared to all the other epidural groups. No significant difference between the five groups was recorded in regard to the first voluntary urination (Table 5). No animals experienced neurological deficits, pruritus, or alopecia within the follow-up period.

## 4. Discussion

This study demonstrated that the injectate volume of LA obtained using the OCL method promoted adequate analgesia that allowed a significant sparing effect of inhalant anaesthetic and systemic opioids requirements. Moreover, it emerged that 0.15 mL for each cm of OCL% is the required volume of LA to cover the metamers involved in a total unilateral mastectomy surgery in dogs.

In groups where morphine (0.1 mg kg^−1^) was added to the volume of ropivacaine calculated using the OCL%, superior perioperative analgesia with a further decrease on systemic opioid requirement during the first 24 h was recorded. In particular, in groups were the lower concentrations of ropivacaine (0.25% and 0.35%) was administered epidurally, a concentration-dependent pattern of motor block offset was noted.

Due to the high variability in body conditions and spinal length among the subjects belonging to the canine species, in the present study, the authors have calculated the volume of LA epidural injected based on the individual vertebral column length; which resulted in an overall mean volume of ropivacaine of about 0.49 mL kg^−1^. The volume used in our study is much higher than the 0.2 mL kg^−1^ [30,31] or the usually recommended without scientific support, of a maximum of 6 mL for dogs weighing more than 30 kg [32].

Nevertheless, volumes higher than 0.3 mL kg^−1^ of lidocaine have been previously investigated in dogs undergoing ovariohysterectomy [33], while clinical dermatomal block at the level of the cervical vertebrae has been reported using bupivacaine 0.25% at a volume of 0.6 mL kg^−1^ [34].

Epidural administration of a high volume of LA has been reported to cause respiratory depression [35,36]; however, in the present study, only 1.6% of dogs that received epidural injection of ropivacaine (4/40), developed hypoventilation (PE’CO_2_ ≥ 50 mmHg (6.6 kPa)). This result is particularly interesting if compared to the incidence of hypoventilation recorded in the control group where mechanical ventilation resulted necessary in all dogs.

In epidural groups, the four dogs that required mechanical ventilation received the higher concentration of ropivacaine (*n* = 2 R_0.5%_ and *n* = 2 MR_0.5%_). It is possible that a correlation between the higher concentration of ropivacaine epidurally injected and the respiratory depression recorded may exist, but further studies are required to investigate this aspect. Mild to severe respiratory depression has been reported by other authors following administration of 0.25% bupivacaine at 0.4 mL kg^−1^ [35,36] and 0.6 mL kg^−1^ [36].

Among factors that play a role in the cephalad distribution of LA within the spinal canal, there is the velocity of the injection. In the present study, all the epidural injections were performed over approximately 4 min. However, a recent study showed that an injection velocity of 1 mL min^−1^ compared with 2 mL min^−1^ of 0.5% bupivacaine has a limited effect on injectate distribution and sensory blockade in anesthetized dogs [37].

Finally, all four dogs that required mechanical ventilation were obese (BCS 7/9); beside the influence of body weight on respiratory function, a greater amount of extradural fat in these subjects can explain a more cranial spread of the LA solution involving higher thoraco-cervical segment [38,39].

The significant sparing effect on the FE’Iso (<1.1%) recorded for all dogs belonging to R_0.5%_ and MR_0.5%_ groups may have reduced further respiratory adverse effects due to isoflurane. Nevertheless, in all other cases (16/20) that received 0.5% ropivacaine, an adequate analgesic plan was recorded without clinical evidence of respiratory impairment.

On the other hand, it is not surprising that all dogs belonging to the control group required mechanical ventilation to manage intraoperative hypercapnia [40]. This could be due to a combined effect of fentanyl and isoflurane on the respiratory system.

In regard to cardiovascular depression, 50% of dogs belonging to the control group required vasoactive support (8 ± 2.1 µg kg^−1^ min^−1^ vs. 5 ± 0 µg kg^−1^ min^−1^) during the intraoperative phase, while only 0.8% of those who received epidural ropivacaine required vasopressors. Among the latter, only those who received a higher concentration of ropivacaine (*n* = 1 in R_0.5%_ and *n* = 1 in MR_0.5%_) required vasopressors. Nevertheless, in the present study, the incidence of cardiovascular depression developed after epidural LA injection was lower than the previously reported incidence of 9% [10]. It is possible that a higher requirement of intraoperative fentanyl recorded in all dogs belonging to the control group elicited an increase in vagal tone through centrally mediated mechanisms leading to a cardiovascular support requirement [40,41].

On the other hand, dose-related cardiovascular depression of inhalation agents such as isoflurane is already known [42,43], and even though opioids have inhalant-sparing effect, the opioid vagal-mediated bradycardia effects produced by fentanyl [25,40] may have partially prevented a greater increase in MAP that would be expected with a reduced inhalant anaesthetic administration [44].

In agreement with previous studies, the presence of morphine 0.1 mg kg^−1^ as an adjuvant to epidural ropivacaine was associated with a decrease of 30% of inhalant requirement and in superior postoperative analgesia, as evidenced by a lower total methadone requirement [10,13]. Albuquerque and colleagues reported a duration of analgesia associated with epidural ropivacaine (0.3 mL kg^−1^ at 0.75%) when combined with 0.1 mg kg^−1^ morphine of about 447 min compared to 375 min when ropivacaine was administered alone [45,46]. In the present study, the analgesia obtained after epidural administration of ropivacaine alone was slightly longer, 7 h (420 min), while a significantly longer duration was recorded when ropivacaine was combined with morphine, 12 h (720 min). The reason for the longer analgesic duration observed in our study compared to Albuquerque’s one could be related to the higher volume (about 0.49 mL kg^−1^ in our study vs. 0.3 mL kg^−1^).

One of the most frequently reported adverse effects of morphine epidural administration is urinary retention for which detrusor muscle relaxation and the consequent decreased contractility of the bladder is blamed [11]. In the present study, the first voluntary urination was recorded two hours earlier in MR_0.35%_ group than in all other groups. Interestingly, the dogs belonging to the control group and the ones belonging to MR_0.5%_ registered similar timing.

In fact, despite an hourly postoperative pain assessment and a rescue analgesic plan, higher pain scores were recorded in all dogs belonging to control group accompanied by uninterrupted vocalization, anxiety, and/or immobility and/or reluctancy to move. It is likely that these events may have influenced the voluntary urination activity of the control group resulting in times similar to the MR_0.5%_.

Differential blockade refers to the ability of the LA to block sensory impulses while sparing motor and proprioception functions. In spite of the fact that the mechanism is not fully understood, and various factors may influence the offset, the motor block provides by LA could be defined as concentration-dependent [47,48]. Differential blockade may explain the dissimilar motor block resolution patterns observed in the present study; motor blockade lasted about 8 h among dogs receiving 0.5% ropivacaine, as reported by previous researchers [44], but the motor block resolution occurred significantly earlier (4h) in dogs receiving 0.35% and 0.25% of ropivacaine.

The present study has some limitations that need to be addressed. First, the study was based on a small sample size for each group, and animals were recruited from the canine patient population seen at one veterinary teaching hospital, which may limit generalization. Additionally, since only female dogs were included, future studies should examine whether the same findings will be obtained in male dogs as well.

Despite efforts to maintain body temperature during surgery, hypothermia developed in almost all cases. It is not possible to exclude that the intraoperative hypothermia recorded influenced the anaesthetic requirement and the cardiovascular and respiratory responses recorded in the present study.

Furthermore, the impact of the epidural injection on the respiratory system was also evaluated/measured only during the surgery, where hypercapnia was recorded as an indicator of respiratory depression. Postoperatively, all dogs were clinically monitored for signs of respiratory disturbance/impairment which did not occur in any of the studied dogs. However, arterial blood gas analysis should be preferred in order to better understand the effect of ropivacaine concentration on the respiratory function. Last, the present study did not include groups where the volume of the LA epidurally injected was calculated on the body weight of the subjects. More studies are certainly needed to compare these two methods of calculating the LA volume to inject epidurally.

Despite epidural anaesthesia being one of the most extensively used techniques for perioperative analgesia/anaesthesia both in human and veterinary medicine, finding the balance between intensity, cranial spread, length of analgesia, and motor block, it still represents a challenge.

The results of this study suggest that the use of OCL to calculate the volume of epidurally injected LA promotes an adequate extension of the block (up to T_1_ in this case) resulting in an approximately constant volume of LA as 0.15 mL cm^−1^ of OCL%.

## 5. Conclusions

In this study, epidural volume of injectate using a dose regimen based on occipito-coccygeal spinal length resulted in a safe and effective methodology to calculate the volume of ropivacaine in bitches undergoing total unilateral mastectomy.

All epidural treatments provided adequate intraoperative analgesia with significant reduction of systemic opioids and inhalant anaesthetic requirements without cardiovascular and respiratory adverse effects.

Postoperatively, in dogs that received epidural anaesthesia, a significant reduction of postoperative pain scores, systemic opioid, anxiety, and fear was recorded. The addition of morphine to ropivacaine reduced postoperative opioid requirement, to almost zero, without negatively affecting the urinary function. The resolution of the motor block was anticipated, without losing analgesic property, in epidural treatments with lower concentrations of the ropivacaine.

## Figures and Tables

**Table 1:** Surgery time points registered during the procedure.

Time	Procedure
S_base_	5 min before skin incision
S_X1_	Skin incision
S_X2_	Subcutaneous dissection
S_X3_	Large veins and artery ligatures
S_X4_	Mammary chain removal
S_X5_	Skin closure

**Table 2:** The animals included in the study (50 female dogs) were divided based on their breeds.

Number	Breed
7	Cross breed > 10 kg
6	Cross breed < 10 kg
5	Beagle
2	Boxer
5	Dachshund
3	German Shepherd
6	Labrador
2	Lagotto
3	Maltese
2	Pointer
1	Miniature Poodle
4	Retriever
2	Rottweiler
1	Standard Poodle
1	Yorkshire terrier

**Table 3:** Demographic data of fifty dogs undergoing unilateral mastectomy randomly allocated in 5 Groups.

Group	Ages (month)	Weight (kg)	OCL (cm)	OCL Range (cm)	LT_1_ (cm)	LT_1_ Range (cm)	OCL% (%)	Injected (mL/cm OCL%)	Injected (mL/kg range)	Injected (Total mL)	Injected (mL kg^−1^)
C	124 ± 15	20.2 ± 11.5	69.4 ± 5	(43–72)	48.8 ± 16	(30–67)	69.3 ± 5	-	-	-	-
R_0.5%_	117 ± 26	21.5 ± 10	65.1 ± 12	(41–78)	46.5 ± 8.9	(29–57)	71.1 ± 1.5	0.16 ± 0.05	(0.2–1)	7.8 ± 2.7	0.48 ± 0.2
MR_0.5%_	123 ± 17	20.5 ± 12.3	63 ± 12.4	(41–83)	45 ± 10	(28–64)	69.5 ± 4.5	0.14 ± 0.1	(0.3–0.9)	8.8 ± 3.4	0.51 ± 0.2
MR_0.35%_	129 ± 16	28 ± 5.8	68.7 ± 7	(49–87)	48.7 ± 23	(25–61)	69.8 ± 1	0.15 ± 0.07	(0.2–1.1)	9.5 ± 2.6	0.5 ± 0.3
MR_0.25%_	113 ± 24	25 ± 7.5	70.9 ± 12	(39–75)	48.8 ± 10	(28–58)	70.1 ± 2	0.15 ± 0.04	(0.25–1)	8.7 ± 1.5	0.49 ± 0.3

Control group (C) no epidural; epidural ropivacaine 0.5% group (R_0.5%_); morphine 0.1 mg kg^−1^ + ropivacaine 0.5% group (MR_0.5%_); morphine 0.1 mg kg^−1^ + ropivacaine 0.35% group (MR_0.35%_); morphine 0.1 mg kg^−1^ + ropivacaine 0.25% group (MR_0.25%_). Occipital-coccygeal length (OCL); the length from the first coccygeal vertebra to the first thoracic vertebra (LT_1_). No statistical differences between groups. Statistical differences (*p*-Value < 0.05).

**Table 4:** Mean values ± standard deviation of heart rate (HR), respiratory rate (*f*R), mean arterial pressure (MAP), end-tidal CO2 (Pe’CO_2_), end-tidal isoflurane (Fe’Iso), and temperature (Temp) for each time point registered during surgery (same time points as Table 1).

Variables	Group	S_base_	S_X1_	S_X2_	S_X3_	S_X4_	S_X5_
HR (bpm)	C	80 ± 19	92 ± 15	65 ± 17	67 ± 19	64 ± 13	68 ± 18
	R_0.5%_	109 ± 8	77 ± 8	70 ± 9	74 ± 5	73 ± 15	72 ± 16
	MR_0.5%_	91 ± 9	88 ± 7	69 ± 5	71 ± 9	75 ± 4	71 ± 9
	MR_0.35%_	88 ± 10	79 ± 7	74 ± 9	80 ± 3	74 ± 8	73 ± 8
	MR_0.25%_	84 ± 13	82 ± 12	77 ± 9	86 ± 12	83 ± 8	86 ± 10
MAP (mmHg)	C	104 ± 18	71 ± 14	66 ± 11	63 ± 8	60 ± 12	60 ± 8
	R_0.5%_	71 ± 16	76 ± 9	74 ± 8	72 ± 12	72 ± 8	78 ± 9
	MR_0.5%_	73 ± 10	65 ± 12	70 ± 8	74 ± 9	69 ± 7	67 ± 8
	MR_0.35%_	81 ± 15	74 ± 7	74 ± 10	79 ± 11	75 ± 9	82 ± 10
	MR_0.25%_	86 ± 10	87 ± 15	86 ± 8	85 ± 7	87 ± 12	86 ± 9
*f*R (brpm)	C	18 ± 5	15 ± 3	15 ± 4	13 ± 2	15 ± 3	10 ± 2
	R_0.5%_	15 ± 5	15 ± 4	17 ± 3	18 ± 6	16 ± 5	18 ± 2
	MR_0.5%_	12 ± 7	14 ± 8	11 ± 6	15 ± 5	12 ± 8	11 ± 6
	MR_0.35%_	17 ± 4	15 ± 6	15 ± 3	13 ± 6	12 ± 3	13 ± 3
	MR_0.25%_	18 ± 5	15 ± 6	17 ± 9	14 ± 5	12 ± 6	14 ± 3
Pe’CO_2_ (mmHg)	C	49 ± 10	39 ± 10	44 ± 3	44 ± 5	45 ± 5	46 ± 6
	R_0.5%_	46 ± 7	41 ± 7	45 ± 8	42 ± 8	39 ± 6	41 ± 7
	MR_0.5%_	45 ± 5	43 ± 7	43 ± 7	44 ± 6	43 ± 8	40 ± 8
	MR_0.35%_	42 ± 8	41 ± 5	42 ± 7	40 ± 6	41 ± 4	43 ± 7
	MR_0.25%_	44 ± 3	43 ± 6	42 ± 9	41 ± 7	40 ± 7	40 ± 7
Fe’Iso (%)	C	1.3 ± 0.05	1.3 ± 0.2 ^†^	1.3 ± 0.15 ^†^	1.3 ± 0.15 ^†^	1.3 ± 0.05 ^†^	1.3 ± 0.15 ^†^
	R_0.5%_	1.3 ± 0.04	1.2 ± 0.04	1.2 ± 0.03	1.1 ± 0.05	1.1 ± 0.05	1.2 ± 0.05
	MR_0.5%_	1.3 ± 0.05	1.1 ± 0.05	1.0 ± 0.05	0.9 ± 0.04	0.9 ± 0.05	0.9 ± 0.05
	MR_0.35%_	1.3 ± 0.03	1.2 ± 0.05	1.1 ± 0.02	1.0 ± 0.03	1.0 ± 0.05	1.0 ± 0.05
	MR_0.25%_	1.3 ± 0.05	1.2 ± 0.05	1.1 ± 0.1	1.1 ± 0.1	1.1 ± 0.05	1.1 ± 0.1
Temp (C°)	C	36.2 ± 0.5	36.2 ± 0.5	36.7 ± 0.3	36.6 ± 0.2	36.8 ± 0.4	36.5 ± 0.9
	R_0.5%_	35.7 ± 0.7	35.4 ± 0.5	35.2 ± 0.5	35.3 ± 0.7	36.2 ± 0.8	36.5 ± 0.5
	MR_0.5%_	35.5 ± 0.8	35.2 ± 0.8	34.8 ± 0.8	34.9 ± 0.8	35.4 ± 0.8	36.6 ± 0.5
	MR_0.35%_	35.9 ± 0.9	35.2 ± 0.7	35 ± 0.9	35.5 ± 0.3	35.8 ± 0.5	36.2 ± 0.4
	MR_0.25%_	35.2 ± 0.7	35.1 ± 0.9	35 ± 0.5	35.5 ± 0.5	35.8 ± 0.5	36.0 ± 0.7
Mean Fentanyl	C	9.8 ± 5.5 µg kg^−1^ h^−1 †^					
	R_0.5%_	1.1 ± 0.5 µg kg^−1^ h^−1^					
	MR_0.5%_	0.2 ± 0.14 µg kg^−1^ h^−1^					
	MR_0.35%_	0.7 ± 0.6 µg kg^−1^ h^−1^					
	MR_0.25%_	1.2 ± 0.9 µg kg^−1^ h^−1^					

Control group (C) no epidural; epidural ropivacaine 0.5% group (R_0.5%_); epidural morphine 0.1 mg kg^−1^ + ropivacaine 0.5% group (MR_0.5%_); epidural morphine 0.1 mg kg^−1^ + ropivacaine 0.35% group (MR_0.35%_); morphine 0.1 mg kg^−1^ + ropivacaine 0.25% group (MR_0.25%_). No statistically significant differences were evidenced between groups for HR, MAP, PE′CO_2_, fR, and temperature values. Statistically significant differences between the C group and all epidural groups for mean FE’Iso values. Fentanyl administration resulted significant higher in C group compared to all other groups. Statistical differences (*p*-Value < 0.05) are reported as (^†^).

**Table 5:** Postoperative evaluation of the locomotor activity (based on the evaluation of the following functions: cutaneous trunci muscle (CTM); patellar reflex, withdrawal response, ability to stand, ability to walk), and voluntary urination, and total methadone requirement during the first 24 h recorded in 50 dogs (5 groups) studied.

Groups	CMT Reflex	Patellar Reflex	Withdrawal Response	Ability to Stand	Ability to Walk	Spontaneous Urination	Methadone over 24 h
C	0.25 ± 0 *	0.25 ± 0 *	0.25 ± 0 *	0.5 ± 0.2 *	0.5 ± 0.7 *	6.9 ± 3.2	1.8 ± 0.5 *
R_0.5%_	6.1 ± 1.2	6.6 ± 1.5	7.1 ± 1.1	7.4 ± 1.3	7.8 ± 1.1	6.1 ± 1.2	0.8 ± 0.1
MR_0.5%_	5.6 ± 1.0	5.3 ± 1.4	5.3 ± 1.2	7.4 ± 1.2	7 ± 1.5	7.6 ± 1	0.0 ± 0.0 *^,^**
MR_0.35%_	3.2 ± 0.5 **	3.7 ± 1.5 **	3.5 ± 0.2 **	3.6 ± 0.7 **	3.8 ± 0.5 **	5.0 ± 1.6	0.3 ± 0.05
MR_0.25%_	3.3 ± 0.6 **	3.5 ± 0.8 **	3.2 ± 1 **	3.5 ± 0.1 **	3.6 ± 0.1 **	5.5 ± 2.8	0.4 ± 0.03

All values are expressed as hours after epidural injection for all groups, excepting for C, where the times reported refer to hours after recovery from general anaesthesia. Methadone requirements during first 24 h are reported as total mg kg^−1^. A significant quicker resumption of the locomotor activity in C group compared to all the other groups was recorded. Within the epidural groups, a statistically significant quicker resumption of locomotor activity was recorded in the MR_0.35%_ and MR_0.25%_ groups compared to the other epidural groups with no significant difference between these two groups. Methadone administered during the first 24 h was significantly higher in C groups compared to all others. Total methadone required was significantly lower in the MR_0.5%_ group compared to all other groups and/or compared between the epidural groups. Statistical differences (*p*-Value < 0.05) are reported as (*) when comparison was made between all groups, and (**) when comparison was done only between epidural groups.

## Data Availability

Data supporting he reported results can be sent to anyone interested by contacting the corresponding author.
